# Perception on blackflies and community ownership of the “Slash-and-Clear” vector control intervention in onchocerciasis-endemic communities along Edo-Ondo border in Southern Nigeria

**DOI:** 10.1371/journal.pone.0344617

**Published:** 2026-03-06

**Authors:** Monsuru Adebayo Adeleke, John Agbonifo, Olabanji Ahmed Surakat, Bertram Nwoke, Kenneth Opara, Hayward Mafuyai, Oluwadamilare Ganiu Dauda, Dauda Ishola Azeez, Olanike Oladipupo, Happy Poko, Francisca Olamiju, Emmanuel Emukah, Chukwuemeka Makata

**Affiliations:** 1 Department of Animal and Environmental Biology, Osun State University, Osogbo, Nigeria; 2 Department of Sociology, Osun State University, Osogbo, Nigeria; 3 Department of Animal and Environmental Biology, Imo State University, Owerri, Nigeria; 4 Department of Animal and Environmental Biology, University of Uyo, Uyo, Nigeria; 5 Department of Zoology, University of Jos, Jos, Nigeria; 6 State NTDs Programme, Ondo State Ministry of Health, Akure, Nigeria; 7 NTDs Programme, Edo State Ministry of Health, Benin City, Nigeria; 8 MITOSATH, Barakin Akawo, Nigeria; 9 The Carter Centre, Nigeria Office, Benin; 10 Neglected Tropical Diseases Unit, Federal Ministry of Health, Abuja, Nigeria; University of Energy and Natural Resources, GHANA

## Abstract

**Background:**

Many communities along the Edo-Ondo border in southern Nigeria remain highly endemic for onchocerciasis. Despite more than two decades of mass drug administration (MDA), transmission persists, necessitating the National Onchocerciasis Elimination Committee (NOEC) to designate the zone as special intervention hotspots. Slash-and-Clear (S&C) has emerged as a promising, community-based approach that can be integrated with ongoing MDA campaigns. This study assessed community knowledge of blackfly, onchocerciasis and willingness to sustain S&C without external financial or logistical support.

**Method:**

Structured questionnaire was administered to 79 key informants from Edo (n = 59) and Ondo (n = 20) communities along Ose River. Focus group discussions (FGDs) were conducted pre- and post-intervention (April 2023 and April 2025, respectively) with participants from 76 communities that participated in slash-and-clear. Three communities declined due to security and socio-cultural reasons.

**Results:**

Prior to intervention, all 79 (100%) key informants reported frequent blackfly bites and expressed the need for strategies to reduce the nuisance. FGD findings corroborated this, with participants recognizing blackflies and their biting burden but showing limited awareness of onchocerciasis as a disease. Common protective measures included wearing long clothing and applying chemical substances on skin to prevent bites. At post-intervention, participants collectively reported noticeable decline in blackfly activity, which reinforced community enthusiasm for sustaining the intervention. Although some communities expressed willingness to continue without monetary incentives, the provision of essential tools and materials was consistently emphasized by majority.

**Conclusion:**

The reduction in blackfly nuisance was a motivator for community’s willingness to sustain the intervention. Continued support and routine monitoring by the Edo and Ondo State governments, especially for communities reluctant to participate without financial incentives, are critical to prevent reinfestation in committed areas. This supervision will ensure the broader success of the S&C strategy and the elimination of onchocerciasis in communities along the Edo-Ondo border.

## Introduction

Onchocerciasis is one of the neglected tropical diseases and remains a major global public health concern, commonly associated with characteristic onchocercal nodules, severe skin disease (onchodermatitis), lymphatic complications (such as hanging groin), epilepsy in children and impaired vision and blindness [1]. In West Africa, blackflies of the *Simulium damnosum* complex have been established as the principal vector of onchocerciasis [2,3]. Blackflies breed in fast flowing, highly-oxygenated rivers or streams and their immature stages can be found attached to rocky substrates, submerged and trailing vegetations. The preference for this specific breeding habitats restricts their presence primarily to communities close to viable breeding sites, which in turn shapes the geographical distribution of onchocerciasis [4]. The distribution, intensity, and endemicity levels of onchocerciasis are largely influenced by the ecology and behaviour of the blackfly, particularly the effective flight range, when seeking bloodmeal (appetitive flight driven by the need to find blood), which is unlikely to exceed 15 km from breeding sites [4]. As a result, the most heavily affected communities are almost invariably located within 15 km radius to the breeding site [4]. This breeding site preference and oviposition behaviour of the blackflies influenced the current intervention being deployed to control or eliminate onchocerciasis in this study.

Nigeria bears the highest burden of onchocerciasis globally, contributing approximately 40% of all reported cases worldwide [5]. The first nation-wide Rapid Epidemiological Mapping of Onchocerciasis (REMO) was conducted between 1990 and 1995 to determine the intensity and distribution of the disease in the country [5]. Results of this mapping survey showed that about 50million people living in over 40000 communities are at risk and the disease is endemic in all states except for Lagos, Rivers, Katsina and Bayelsa states [5]. The World Health Organization (WHO) recommends mass drug administration (MDA) with ivermectin as the primary strategy for controlling onchocerciasis [6]. In Nigeria, this approach has been implemented for over two decades, with States progressing at different stages of intervention [5]. However, despite the successes recorded with the use of ivermectin, there are still pockets of unabated transmission in some endemic foci.

REMO result showed that both Ondo and Edo States are endemic for onchocerciasis [5]. Edo State is designated as a highly endemic area for onchocerciasis, especially in five local government areas in the western part of the state that border Ondo State [7]. This region has consistently reported persistent transmission, likely linked to inadequate coverage of mass drug administration efforts [8,9]. Considering the flight range behaviour of the vector, this unabated transmission increases the risk of cross-border transmission of onchocerciasis along the Edo-Ondo border. To achieve the elimination of transmission of onchocerciasis along the Edo-Ondo border, there is a need to complement MDA with vector control intervention. This complementary strategy has been recommended to accelerate onchocerciasis elimination in hotspot areas in a timely manner [10]. The effectiveness of MDA and vector control using larvicides at breeding sites has been reported to result in the elimination of transmission in 15 out of 17 endemic foci in Uganda [11–13]. Similar outcomes were also observed on Bioko Island in Equatorial Guinea where this complementary strategy led to transmission interruption [14]. Despite the effectiveness of larviciding, concerns remain regarding its cost, environmental impact, and the need for skilled personnel to ensure accurate dosing and safe application. This limitation of larviciding highlights the need for alternative vector control strategy.

Slash-and-Clear (S&C) is a cost-effective, community-driven, and environmentally sustainable strategy that has proven successful in significantly lowering blackfly populations and reducing the transmission of *Onchocerca volvulus* [15]. The S&C intervention have been found to result in over 90% reduction in the biting rates of blackflies for over 3 months [15] and up to 6 months [16]. These results suggest that for slash-and-clear to effectively interrupt transmission, it must be implemented consistently and repeatedly. The persistent transmission along the Edo-Ondo border highlights the importance of complementing ivermectin distribution with the S&C intervention and ensuring it is community-driven to ensure long-term sustainability. Hence, the pre-intervention phase was designed to assess community knowledge and perceptions of blackflies, determine whether residents recognized the need for vector control, and evaluate their willingness to implement the intervention to reduce blackfly populations locally. The post-intervention phase focused on assessing community perceptions of the effectiveness of S&C, identifying challenges to its implementation, and determining the extent to which communities were prepared to assume ownership of the intervention by providing the necessary financial and logistical support without reliance on external funding, as part of efforts to strengthen ongoing elimination programs.

## Materials and methods

### Ethical approval

The study protocol was approved by the National Health Research Ethical Committee of the Federal Ministry of Health, Nigeria (NHREC/01/01/2007–13/03/2023). The Federal Ministry of Health (NTDs Units), the State Coordinators of the NTDs and the community leaders in the study communities were mobilized for the study. Participants were recruited for the study between April 2023 and April 2025, and their participation in the questionnaire and focus group discussions was entirely voluntary. Written informed consent was obtained from all participants after the objectives, benefits, risks and confidentiality protocols had been duly explained and understood by them. Audio recordings were securely stored and accessed only by the social and behavioural scientists for transcription. All personal information was removed during transcription to maintain participant confidentiality.

### Study area

**Fig 1:**
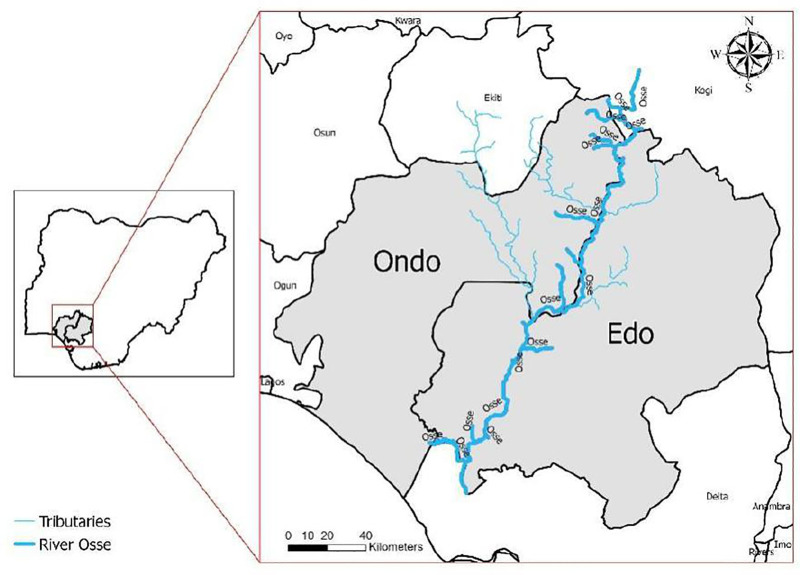
Map of Edo-Ondo border with the river system.

This study was conducted in 79 communities along the Edo-Ondo border, including 59 in Edo and 20 in Ondo. Ose river and its tributaries transverse the communities along the border of the two states (Fig 1). The slash-and-clear intervention was implemented in 58 communities within five Local Government Areas (LGAs) in Edo State (Owan West, Ovia Northeast, Ovia Southwest, Akoko Edo, and Owan East) and in 18 communities within four LGAs in Ondo State (Ose, Owo, Akoko South-East, and Idanre). The remaining three communities declined in conducting S&C due to insecurity and other socio-cultural reasons. In Edo region, two intervention communities declined to participate attributing the S&C as a destructive way to the livelihood of the residents. The communities are fishing communities and claimed that the vegetation serves as source of food to the fish in the river upon which the socio-economic fortune of the residents depends on. In Ondo region, three intervention communities could not implement the intervention as a result of security challenges in the area. These Edo-Ondo border communities have been previously documented as hotspots for onchocerciasis, with evidence of ongoing transmission despite more than two decades of annual mass drug administration (MDA) with ivermectin [8,9,17,18]. Although MDA coverage has generally been high along the Edo–Ondo border over the past 4–5 years following the introduction of biannual ivermectin distribution, cases of persistent transmission have been reported, leading the National Onchocerciasis Elimination Committee (NOEC) of the Federal Ministry of Health to designate the Edo–Ondo border communities as a special intervention zone or hotspot [19]. The main sources of livelihood in these communities are fishing and agriculture, including both subsistence farming and cash crop cultivation, particularly cocoa. Edo and Ondo States typically experience two distinct seasons; the wet season (April to October) and the dry season (November to March).

The administrative boundary shapefile was obtained from geoBoundaries: a global database of political administrative boundaries available at https://www.geoboundaries.org. The Ose River system shapefile was obtained from AQUASTAT (FAO) Rivers of Africa dataset available at https://data.apps.fao.org/catalog/iso/b891ca64-4cd4-4efd-a7ca-b386e98d52e8. Both datasets are provided under the Creative Commons Attribution 4.0 International (CC BY 4.0) license.

### Study design

This was a community-based mixed-methods study comprising a cross-sectional quantitative survey using a simple questionnaire administered to selected key informants from each of the 79 communities along the Ose River and its tributaries on the Edo–Ondo border to obtain information on blackfly biting rates, alongside qualitative data collected through focus group discussions. One key informant was recruited from each community. Two rounds of focus group discussions (FGDs) were also conducted with participants from the communities that participated in slash-and-clear (76 communities). Participants were purposively selected from each community. Recruitment was facilitated by the State NTD team in collaboration with designated community focal persons, who assisted in identifying eligible individuals based on age, length of residence, and involvement in community activities. Only adults aged ≥18 years who had resided in the community for more than five years were included. The pre-intervention FGD was conducted in April-May 2024 while the post-intervention phase was carried out between April and May, 2025. The FGD at pre-intervention phase was deployed to determine community knowledge on blackflies and assess their level of preparedness and willingness to sustain the intervention. The FGD at post-intervention was used to assess the community’s assessment of the effectiveness of the S&C intervention, identify challenges and communities’ and willingness to sustain the S&C intervention in their respective communities without external logistical and financial support towards ownership and sustainability.

### Study participants

The State teams for Neglected Tropical Diseases (NTD) in Edo and Ondo facilitated initial community entry across all selected villages. These visits involved meetings with community leaders to explain the study objectives, which included assessing blackfly presence, understanding the onchocerciasis situation in their communities, and implementing the slash-and-clear intervention. Community heads and three adult residents, including at least one female representative from each community to ensure inclusivity, were invited to the pre- and post-intervention meetings held separately in each state. During the meetings, the purpose of the intervention and the focus group discussion procedures were explained, including the intention to audio-record the sessions. Training sessions and educational videos on blackfly bioecology, seasonal abundance, and slash-and-clear activities were also presented prior to the focus group discussions. A paper-based questionnaire was deployed to 79 key informants, one from each community to assess their knowledge on biting nuisance of the blackflies and their need for strategy to control the flies. Additionally, each focus group was randomly composed, ensuring that each LGA had at least one representative. There were 7–10 participants in each FGD. In both the pre- and post-intervention sessions, participants were seated around a table, and audio recording commenced following a brief reiteration of the study purpose. Each session was facilitated by four trained social and behavioural scientists who guided the discussion and ensured responses remained relevant to the study objectives. Although the discussions were conducted in English, interpreters were available to aid communication for participants more comfortable speaking in local languages, ensuring both comprehension of the questions and accurate relay of participant responses. At the conclusion of each focus group discussion, the research team expressed their gratitude to the participants and addressed any remaining questions or concerns.

### The slash-and-clear intervention

In each selected intervention community, five young men aged 18 years and above, who voluntarily consented and were knowledgeable about the river terrain, primarily fishermen, were recruited by community leaders to implement the S&C intervention. The recruits were taken to identified blackfly breeding sites located upstream and downstream of the communities and trained to cut and remove trailing and submerged vegetation from the river. On designated S&C days, the recruits entered the river, and with the use of the cutlasses provided, they slashed trailing and submerged vegetations, which was subsequently cleared from the river and discarded on the riverbanks to dry, thereby destroying attached larvae and pupae. Slash-and-clear activities covered a 1 km stretch upstream and downstream of each intervention site. The S&C activities lasted between three and five days, depending on the specific characteristics of the breeding sites in each community. Communities were provided with logistical and financial support to facilitate the slash-and-clear activities.

### Data analysis

Questionnaire data were entered into Microsoft Excel 2021, where descriptive analyses and frequency distributions were generated. Recordings from the FGDs were transcribed verbatim by the social and behavioural science team. The transcripts were then subjected to thematic analysis. Members of the research team carefully reviewed the transcripts, identified recurring patterns, and developed codes under different themes to systematically regroup participants’ views. Relevant data were summarized within each code and organized into thematic categories that reflected the study objectives, including community perceptions of blackfly nuisance, views on the slash-and-clear intervention and its effectiveness, willingness to sustain the intervention with no or minimal external support, as well as implementation challenges.

## Results

### Baseline information obtained from key informants

All 79 (100%) key informants acknowledged the nuisance caused by blackfly bites. In Edo, 79.66% reported itching as a major effect, while 85.0% reported itching as symptom in Ondo. All key informants from the 79 communities (100%) expressed the need for strategies to reduce blackfly population in their communities (Table 1).

**Table 1:** Knowledge on the biting nuisance of blackflies at the study area based on questionnaire data.

Question	Response	Edo (%)	Ondo (%)
Do blackflies bite in your community?	Yes	59 (100.00)	20 (100.00)
	No	0 (0.00)	0 (0.00.)
How often?	Very often	50 (84.74)	18 (90.00)
	Occasionally	9 (15.26)	2(10.00)
What are the effects from blackfly bite?	Itching	47 (79.66)	17 (85.00)
	Swelling	12 (20.33)	3 (15.00)
	No idea	0 (0.00)	0 (0.00)
Do you need strategy to reduce the bite?	Yes	59 (100.00)	20 (100.00)
	No	0 (0.00)	0 (0.00)

### Pre-intervention FGD

The FGD findings are presented under five key themes derived from participant responses: perceptions of blackflies and onchocerciasis, community response and coping strategies, barriers to sustained implementation, community cohesion and motivation, and feasibility of intervention.

### Perceptions of blackflies and onchocerciasis

Participants across Edo and Ondo States recognized blackflies as a serious nuisance, with bites described as painful and resulting in long-lasting discomfort. Although most respondents had no knowledge of the term “onchocerciasis,” they were familiar with the symptoms associated with blackflies bites which include severe itching and skin depigmentation, referring to blackflies as insects whose bites caused “craw-craw.”

### Community response and coping strategies

When prompted on preventive measures, majority of the participants have developed adaptive strategies to cope with their presence. Participants described applying “palm oil” to exposed skin and “wearing thick clothing” to reduce bites, enduring the “pain and discomfort” as an inevitable part of their lives.

### Barriers to sustained implementation

When participants were asked to identify barriers to implementing the intervention, participants identified several challenges. These include the physical difficulty of removing big vegetation in rivers, intra-communal tensions, and competing economic interests. For instance, participants from some communities such as Kekere and Siloko community in Ovia Southwest noted that fishing practices such as grass planting by Ijaw fishing communities along the rivers could present a challenge, as these fishermen might oppose the removal of these vegetations.

### Community cohesion and motivation

Trust deficits and leadership conflicts were cited as barriers to collective action. Participants stressed the need for community sensitization, unity, and transparency to ensure participation of community members. They emphasized that motivation, especially through monetary incentives and proper awareness campaigns, would be essential to mobilize participation, particularly among youths since all of them are either fishermen or farmers. Their participation in S&C will require them abandoning the farms for days and there must be motivation from a quarter. Concerns were raised about possibility of rancour if incentives were not transparently managed by the head of the community.

### Feasibility of intervention

Participants collectively expressed optimism for the S&C approach, opinions varied regarding the timing. Overall, participants supported the intervention but emphasized that sustained success would require local ownership and appropriate incentives.

### Post-intervention FGD

The post-intervention findings are presented under five key themes: participation and community engagement, effectiveness of intervention and community motivation, implementation challenges, community willingness to sustain intervention and required support; and recommendations for sustainability.

### Participation and community engagement

When the community members were asked if they all carried out the intervention in their communities, 58 (98.30%) out of the 59 communities enrolled in Edo State participated in the exercise. The participants from Gelegele remarked that their non-participation was that “*The fishermen in the community didn’t agree with the cutting of the vegetation in the river, as they claimed it is important to their fishing activities*..” In Ondo State, 18 (90%) of 20 selected communities participated. The remaining two communities did not participate as a result of insecurity and communal clashes in the area. In general, willingness to participate was high, but faced some challenges, including resistance from fishermen who do not want the vegetations to be removed for economic reasons and there are also concerns that some heads of communities have a habit of personalizing the cutlasses and other tools provided by the researchers.

### Implementation challenges

The challenges encountered during the S&C intervention were broadly categorized as logistical, environmental, and social. Logistically, several communities faced shortages of essential tools such as cutlasses, gloves, rainboots, and sharpening files, along with limited transportation for supervision, particularly motorcycles. Environmentally, access to breeding sites was hindered by dense vegetation, high river levels during the rainy season, and swampy riverbanks. Socially, resistance from some community members especially fishermen who rely on the vegetation in the rivers for their fishing activities.

*Ipese community, Akoko Southeast, Ondo State: “Transportation was a challenge as breeding site was long and far to the community”*

However, despite some of these challenges, some of the communities were able to still implement the S&C after intervention by community leaders.

*Kekere and Siloko community, Ovia Southwest, Edo State: “There was resistance from fishermen who initially opposed the intervention until community leaders intervened and ordered for the slash-and-clear be done”.*

### Effectiveness of intervention and community motivation

Across both states, respondents collectively reported notable decline in blackfly density and biting nuisance following the intervention. Some communities noted visible improvement in children's skin health, suggesting potential impact on onchocerciasis symptoms. A respondent from Idogun, Ose LGA claimed “*There was drastic reduction. Many children used to have bite rash on the body but we noticed a reduction in the rash and itching after slash-and-clear*”. Despite the perceived health benefit, some communities still linked their motivation to financial incentives, suggesting that while perceived benefit matters, logistical and financial support remains a critical motivation since they are farmers and they need to abandoned their works for days. However, some communities namely Ulorin, Iguariakha, and Ugbogui in Ovia Southwest; Lakaloko in Owan Southwest; Okenew and Atoruru in Owan West all in Edo region; and Oke-Odo in Owo LGA, Owani and Sonel Bonel communities in Ose, Ondo State expressed willingness to sustain the intervention. According to participants in Oke-Odo, their motivation stemmed from the “*visible reduction in blackfly bites and health benefits*” observed following the previous round of the S&C intervention.

### Willingness to sustain intervention and required support

While some communities in Edo State expressed willingness to continue the intervention without financial incentives, most communities in Ondo and a few in Edo indicated that their continued participation would depend on the provision of non-monetary logistical support. These communities specifically requested for more items such as cutlasses, chainsaws, rainboots, motor-saws, and transportation to facilitate the intervention. They also emphasized the need for ongoing community sensitization to enhance awareness of blackfly control and the importance of the intervention. Additionally, improved supervision and follow-up were highlighted as very important, with some participants recommending the establishment of local supervisory committees to ensure that tools are not personalized.

*Igbogui community, Ovia Southwest, Edo State: “Community members will participate if the community leaders order them to do it even without money.”*

*Atoruru community, Owan West, Edo State: “We will be willing to continue without monetary support.”*

*Oke-Odo, Owo, Ondo State: “If our people have more cutlass and other logistics, they will carry out the intervention once the community head gives the order. The motivation is a result of the health benefit having seen the result of the previous intervention.”*

### Recommendations for sustainability

Although a few communities expressed a need for financial support to sustain the intervention, most participants emphasized that continued logistical support is crucial. It was also unanimously recommended that community leaders should be actively involved, with all materials entrusted to them for safekeeping and future use, to prevent misuse or appropriation by individuals. A respondent from Eti-Ose, Akoko Southeast, noted, “*Supervisors should go to communities to ensure that the slash-and-clear activity is carried out in every community*.” A participant from Ayanran community, Akoko Edo, stated, “*There should be improved community advocacy on the benefits of slash-and-clear and health education for community members so they accept the slash-and-clear*.” Another respondent from Idogun community, Ose LGA, added, “*There should be feedback mechanism where community members can send report and evidences of their slash-and-clear activities. Mectizan program should be implemented annually as it is very good based on personal experience*.”

## Discussion

This study highlights participants’ understanding of blackfly biting nuisance, their existing behavioural adaptations to blackfly bites, experiences with the S&C intervention, and key challenges and recommendations for sustaining the strategy. Findings revealed that community members were well aware of the intense biting nuisance from blackflies and its consequences. Although most participants were unfamiliar with the term onchocerciasis, they identified symptoms such as severe itching and skin depigmentation as consequences of blackfly bites. These observations align with previous studies conducted in Nigeria [20,21] and Cameroon [22]. To protect themselves, participants reported wearing long clothing and socks, as well as applying chemical substances from local herbs to the skin. Similar practices have been documented in other endemic settings [22–24]. However, during hot weather, particularly when temperatures exceed 26°C, heavy clothing becomes uncomfortable. Additionally, while chemical repellents may offer some protection, they can also pose health risks. Reports of adverse effects such as headaches, respiratory discomfort, skin irritation, and burns have been associated with these substances [22]. Given that participants were provided with training and educational videos on the bioecology and seasonal abundance of blackflies before the FGD, this study did not assess their knowledge of blackfly biology and seasonality.

Participants from communities across both Edo and Ondo States expressed a strong willingness to eliminate blackflies, motivated by their direct experiences with blackfly nuisance and the debilitating effects of onchocerciasis. This aligns with reports that community perceptions of disease burden significantly influence participation in control strategies, as similar enthusiasm was observed in Cameroon [25]. Despite this motivation, several challenges persist. Logistical barriers, such as limited access to essential tools and difficult river terrains, especially during the rainy season when the water levels are high, are some of the challenges highlighted that could impact implementation. Social and economic issues, such as insecurity, lack of youth engagement due to absence of financial incentives, and resistance from fishermen who cultivate crops in river areas, further complicate sustained intervention efforts. Some of these obstacles have been identified in other regions, including the Ntui district in Cameroon [25].

Interestingly, some communities, particularly in both Edo and Ondo states, despite the challenges demonstrated a willingness to sustain the intervention even in the absence of monetary incentives. This reflects the value placed on the observed effectiveness of the S&C and health benefits and the potential for the intervention to significantly reduce the population of blackflies and transmission of *Onchocerca volvulus* and ultimately achieve elimination of the disease in the communities when implementation is sustained. The impact of S&C on blackfly populations has been well documented, with studies reporting over 50% reduction in blackfly density [26], a 90% decline in biting rates sustained for more than three months [15] and lasting up to six months [16]. Similar expressions of commitment after evident reduction in blackfly populations have been reported in Cameroon [22,25] and South Sudan [3]. Nevertheless, the long-term sustainability of the S&C strategy in many of the communities depends on continued support in the form of provision of basic tools, community sensitization, and routine supervision as unanimously stated by participants. Another common recommendation by participants was the involvement of community leaders in the planning and oversight of future S&C to ensure accountability and prevent mismanagement of resources. Their inclusion is critical to fostering community ownership and sustaining efforts to interrupt onchocerciasis transmission across the Edo-Ondo border communities.

This study has a few limitations. The provision of logistical and financial support may have influenced participants’ responses, as they might have been reluctant to provide negative feedback. Additionally, long stretches of river with dense vegetation in inaccessible areas limit the reach of the S&C intervention. Some smaller communities along the river were also not included in the intervention, as pointed out by a respondent. These limitations could reduce the overall reach of the intervention, making blackfly population reduction less significant in certain locations. Therefore, achieving optimal treatment coverage during MDA campaigns, as recommended by WHO, remains essential. Additionally, optimizing S&C intervention by implementing during the dry season, when water levels and flow are low.

## Conclusion

The persistent transmission of onchocerciasis in communities along the Edo-Ondo border calls for urgent need for vector control measures to complement ongoing treatment efforts. The demonstrated effectiveness of the S&C intervention led to a strong willingness among several communities to sustain the intervention. To encourage the communities and to ensure sustainability, state governments are encouraged to provide consistent logistical support. Additionally, routine monitoring by the Edo and Ondo State governments, especially to communities reluctant to participate without financial incentives, are critical to prevent reinfestation of blackflies in committed areas in order to ensure the broader success of the S&C strategy and the elimination of onchocerciasis in communities along the Edo-Ondo border.

## Supporting information

S1 File Pre-intervention questionnaire deployed to determine the biting nuisance of blackflies in communities. (DOCX)

S2 File Pre and post intervention FGD deployed to determine the participation in slash and clear and their willingness to sustain the intervention. (DOCX)
