# Supplementary material for: Perception on blackflies and community ownership of the “Slash-and-Clear” vector control intervention in onchocerciasis-endemic communities along Edo-Ondo border in Southern Nigeria
Source: PLoS One. 2026 Mar 6;21(3):e0344617. doi: 10.1371/journal.pone.0344617 (PMC12965575; doi:10.1371/journal.pone.0344617)
Supplement: S1 File — (DOCX) [file pone.0344617.s001.docx]

**Questionnaire on the study “Perception on blackflies and community ownership of the “Slash-and-Clear” vector control intervention in onchocerciasis-endemic communities along Edo-Ondo border in Southern Nigeria”.**

**Research Instrument: Questionnaire Guide**

**Introduction to the Study**

Welcome the participants and provide a brief introduction to the study. Explain the purpose of the research and emphasize the value of their contributions to the success of the Slash and Clear project. Inform them that the discussion will be voice-recorded and that they will be required to sign a consent form. Assure them that all recordings will be treated with strict confidentiality, and that the discussion will follow global ethical standards. Make it clear that participation is voluntary, and they may withdraw at any point without any consequence.

**Section A: Demographic Information**

Name of Participant:

Gender:

Occupation:

Community:

Your Role in Community:

**Section B: Questions on blackflies biting nuisance**

**1. Do blackflies bite in your community? A. Yes B. No**

**2. How often? A. Very often B. Occassionally**

**3. What are the effects from blackfly bite? A. Itching B. Swelling C. No Idea**

**4. Do you need strategy to reduce the bite? A. Yes B. No**
