# Supplementary material for: Perception on blackflies and community ownership of the “Slash-and-Clear” vector control intervention in onchocerciasis-endemic communities along Edo-Ondo border in Southern Nigeria
Source: PLoS One. 2026 Mar 6;21(3):e0344617. doi: 10.1371/journal.pone.0344617 (PMC12965575; doi:10.1371/journal.pone.0344617)
Supplement: S2 File — (DOCX) [file pone.0344617.s002.docx]

**PRE-INTEVENTION FOCUS GROUP DISCUSSION**

**Research Instrument: Questionnaire Guide**

**Section A: Introduction and Background Information**

**Introduction to the Study**

Welcome the participants and provide a brief introduction to the study. Explain the purpose of the research and emphasize the value of their contributions to the success of the Slash and Clear project. Inform them that the discussion will be voice-recorded and that they will be required to sign a consent form. Assure them that all recordings will be treated with strict confidentiality, and that the discussion will follow global ethical standards. Make it clear that participation is voluntary, and they may withdraw at any point without any consequence.

**Section A: Demographic Information**

Name of Participant:

Gender:

Age:

Occupation:

Community:

Your Role in Community:

**Section B: Knowledge and Perception Assessment**

**Knowledge about Onchocerciasis and Black Flies**

1. Have you heard of the word Onchocerciasis?
2. If yes, what does it mean?
3. Can you tell us what you know about black flies?
4. How do you feel about black flies?
5. Do you see black flies as a problem?
6. If yes, why do you consider black flies as a problem?
7. How do you cope with black flies biting?
8. Is living with black flies a normal part of your life?
9. Is it possible to eliminate black flies?
10. If No, why do you say so?
11. What is the general belief about black flies in your family?
12. What is the general belief about black flies in your community?
13. Can you tell us what your elders and grandparents taught you about black flies?
14. What are the effects of onchocerciasis and black fly bites on your social and economic life
15. Black flies breed on the grasses in the rivers and streams that flow through your community. Would it be okay to cut the grasses on the river?
16. Do the grasses have any benefit for anyone in the community?

**Section C: Rapid Assessment Procedures (RAP)**

Stakeholder Engagement

1. Has the community made efforts to eliminate black flies in the past?
2. What efforts has the community made to eliminate black flies from the community?
3. Have the government and NGOs made efforts to reduce black flies biting in your community?
4. Were the efforts successful?
5. If No, why did the attempts fail?
6. Are you doing anything about the black flies presently?
7. If No, why are you not doing something about it?
8. What do you think causes river blindness?
9. Is black flies a curse of the gods?
10. Do you think people should be bothered about black flies biting?
11. Who do you consult to deal with health problems caused by black flies?
12. How do you treat health problems related to black flies?
13. Between traditional medicine and western medicine, which is the more effective treatment for black flies?
14. Is there any conflict between some people or groups in your community?
15. If Yes, tell us about the conflict.
16. Do problems between peoples or groups usually prevent some people from participating in community activities?

**Slash and Clear**

1. Have you heard about slash and clear?
2. If Yes, have you ever participated in any slash and clear exercise?
3. What was the outcome of the slash and clear exercise?
4. Do you think slash and clear can eliminate black flies?
5. Will you be willing to participate in slash and clear?
6. If I tell you that slash and clear can eliminate black flies, will you participate willingly?
7. What usually stop some people from participating in community events like slash and clear?
8. What can be done to make everyone participate willingly?
9. What can be done so that people will participate regularly in slash and clear?
10. Do people trust each other in your community?
11. If No, why is there distrust?
12. What day of the week is culturally feasible for cutting river vegetation?

**Section D: Engagement and Training**

Training and Awareness

1. Have you attended any training sessions related to the Slash and Clear project?
2. If Yes, what did you learn about black flies?
3. What did you learn about the benefits of Slash and Clear?
4. Do you know the benefits of eliminating black flies?
5. If Yes, what are the benefits?
6. Have you ever used the drug, ivermectin?
7. If Yes, do you think the drug is effective and useful?
8. How can we motivate everybody to participate in eliminating blackflies rom your community?
9. In what ways can you motivate yourselves to participate in slash and clear even when we are not around?
10. Can the community do something like slash and clear for themselves without outsiders help?
11. If No, please explain why?
12. What has local health authorities done in relation to mobilise communities for slash and clear exercise?
13. How can local health authorities mobilize communities for Slash and Clear after the project lifespan?
14. What challenges could undermine efforts to mobilise communities for slash and clear?

Appreciation

Thank participants for their time and valuable input.

Note: The questionnaire should be administered in a structured interview format to gather detailed and qualitative responses from participants.

**POST-INTERVENTION FOCUS GROUP DISCUSSION**

**Research Instrument: Questionnaire Guide**

**Section A: Introduction and Background Information**

**Introduction to the Study**

Welcome the participants and provide a brief introduction to the study. Explain the purpose of the research and emphasize the value of their contributions to the success of the Slash and Clear project. Inform them that the discussion will be voice-recorded and that they will be required to sign a consent form. Assure them that all recordings will be treated with strict confidentiality, and that the discussion will follow global ethical standards. Make it clear that participation is voluntary, and they may withdraw at any point without any consequence.

**Section A: Demographic Information**

Name of Participant:

Gender:

Age:

Occupation:

Community:

Your Role in Community:

**Section B: Participation and community ownership**

Question 1: How many communities participated in the "slash and clear" initiative last year? Did your community participate? Please specify any communities that did not engage, and provide the reasons for their non-participation.

Question 2: What were the primary challenges faced by communities in implementing the "slash and clear" exercise? Were there logistical, social, or environmental factors that hindered successful execution?

Question 3: Did the communities observe any reduction in the bite rate of blackflies following the "slash and clear" activities? If so, can you describe the extent of the observed change, and were there any long-term effects?

Question 4: Are the communities willing to repeat the "slash and clear" initiative this year, assuming logistical support such as provision of machetes is available, but without monetary compensation or supervision? What motivat1es this willingness—community health concerns, commitment to the cause, or other factors?

Question 5: What types of non-monetary support (e.g., provision of materials, training, or community sensitization) would be helpful to sustain the "slash and clear" activities in the future, without any financial compensation?

Question 6: Was there any resistance from community members during the "slash and clear" process in the communities that participated? If resistance occurred, how was it addressed and resolved?

Question 7: Are the communities willing to continue the "slash and clear" process on an annual basis with minimal logistical support? If so, what challenges do you foresee in sustaining this initiative over the long term, and how can these challenges be mitigated?

Question 8: Do you have any advice or recommendations for the team in terms of improving the implementation of the "slash and clear" process, increasing community engagement, or overcoming barriers to success?

Appreciation

Thank participants for their time and valuable input.
